# Supplementary material for: Fibular notch morphometry and its clinical importance on dry bones
Source: PLoS One. 2024 Aug 13;19(8):e0307387. doi: 10.1371/journal.pone.0307387 (PMC11321577; doi:10.1371/journal.pone.0307387)
Supplement: Attachment — Submitted filename: Reviewers.docx [file pone.0307387.s002.docx]

Reviewers’ comments

1. In discussion the authors have mentioned what other got but not comparted their results with his results.
2. In figure 1 all measurements are not seen like angles etc. **(AFD: from a to c; PFD: from b to c; AAPF: angle between a-c and bc; AASIL: angle between intertubercular line and anterior surface tangent line.) Show this correctly.**
