# Supplementary material for: Fibular notch morphometry and its clinical importance on dry bones
Source: PLoS One. 2024 Aug 13;19(8):e0307387. doi: 10.1371/journal.pone.0307387 (PMC11321577; doi:10.1371/journal.pone.0307387)
Supplement: Attachment — Submitted filename: Response to Reviewers.docx [file pone.0307387.s003.docx]

Responses for Journal Requirements:

1. The requested checks completed and amandments about PLOS ONE's style requirement have been made.
2. All information about this data uploaded as supplementary information tool.
3. An additional information regarding the specimens used in your study added in our manuscript.
4. All information about this data uploaded as supplementary information tool.

Responses for Reviewer 1:

1. In accordance with the reviewer’ suggestion, authors were added comparisons with other studies in discussion part.
2. All measurements in Figure 1 have been corrected. New clinically relevant information were added to our study.

Responses for Reviewer 2:

1. The photos of tibia added instead of hand drawn images in line with the recommendations.
2. Below the Table1 the full form of various parameters is given for easy interpretation.
3. Additional table (Table 2) has been added for clearly understanding of analysis.
4. The relevant latest references have been added.
5. As mentioned in the first sentence of materials and methods section “This study was carried out with 76 dry adult tibial bone specimens (right 38, left 38) with unknown age and sex collected from the Department of Anatomy, Cukurova University, Adana.”.
6. Detailed information about the ImageJ Software and precision point were added in the manuscript. And also for additional information web address of the software were added in the manuscript.
7. Aim & objectives were updated for explaining clearly with the interpretations.

**I would like to thank the editors and reviewers for their valuable contributions.**
